# Well-dispersed Pd nanoparticles on porous ZnO nanoplates *via* surface ion exchange for chlorobenzene-selective sensor†

**DOI:** 10.1039/c9ra09705h

**Published:** 2019-12-20

**Authors:** Zhenyu Feng, Cuiling Gao, Xicheng Ma, Jinhua Zhan

**Affiliations:** Key Laboratory for Colloid and Interface Chemistry of Education Ministry, School of Chemistry and Chemical Engineering, Shandong University Jinan Shandong 250100 P. R. China fengzhenyu@sdu.edu.cn +86 53188363768; Shandong Institute for Product Quality Inspection Jinan Shandong 250102 P. R. China

## Abstract

The extensive use of chlorobenzene in chemical, pharmaceutical, and agrochemical industries poses a severe health hazard to human beings, because it is highly toxic. The detection of chlorobenzene by metal oxide gas sensors is difficult, owing to its chemically inert molecular structure. In this study, well-dispersed Pd nanoparticles were deposited on porous ZnO nanoplates *via* surface ion exchange, followed by H_2_ reduction. The preparation process effectively prevented the aggregation and uncontrollable growth of Pd particles. A gas-sensing test was conducted, and the modification of size-controlled Pd nanoparticles was found to effectively enhance the sensing properties of porous ZnO nanoplates to chlorobenzene over 300 °C (higher sensitivity at a low operating temperature). At 440 °C, 5% Pd@ZnO sensor showed a drastic increase in response by nearly 4.5-fold, as well as excellent sensing selectivity to chlorobenzene. Its repeatability and stability were acceptable. As known, Pd nanocatalysts contribute to the oxidation of chlorinated aromatic compounds. Pd@ZnO sensors generated more catalytic sites and oxygen species (confirmed by XPS), thus enhancing chlorobenzene oxidation and improving the sensitivity of ZnO-based gas sensors.

## Abstract 2

Well-dispersed and size-controlled Pd nanocatalysts were deposited on porous ZnO nanoplates *via* surface ion exchange for enhanced and selective chlorobenzene-sensor.

## Graphical abstract

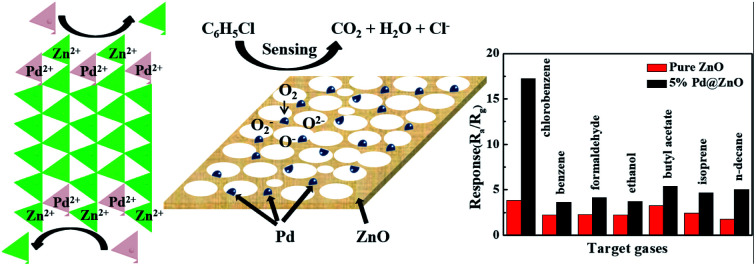

## Introduction

Chlorobenzene has widespread applications in chemical and pharmaceutical industries. It is a highly toxic and health-threatening chemical.^1^ Its detection is important for maintaining standard environmental conditions and avoiding environmental hazards. Gas chromatography is the generally routine technique utilized for chlorobenzene detection.^2^ However, rapid on-site monitoring of VOCs (volatile organic chemicals) free from pretreatment is pursued.^3^ Gas-sensing technology has these advantages. Gas detection by semiconductors is a method that converts electrons into electrical signals by redox reaction of target gas molecules on the surfaces of gas sensors.^4^ The simple electronic gas-sensing system is prominent candidate for use in chlorobenzene detection.^5^ The development of gas-sensitive materials is the key, and the research on metal-oxide-based gas sensors has attracted much attention.^6^

ZnO has been widely used in the past few decades as a well-known gas-sensing material. It is an n-type semiconductor with a large excitation energy and remarkable photonic and electronic properties.^7^ The material engineering of ZnO to adjust and control its morphology and structure has been conducted to improve its sensing properties.^8–14^ Porous ZnO nanoplates have a larger specific surface area with large number of active sites, which results in its better sensing performance.^13^ Because of the limitations of the intrinsic properties of ZnO, various composite structures based on porous ZnO nanoplates have been fabricated. These can behave as high-performance gas sensors. Noble metals (*e.g.*, Au, Pt, and Pd), which behave as sensitizers, have been often used to modify gas-sensing materials such as semiconductor oxides.^15–17^ Recently, the sensors based on ZnO nanowires decorated with Pd nanoparticles have also been reported, which obtain high selectivity and sensitivity.^18,19^

The poor response and low sensing selectivity of metal-oxide gas sensors towards chlorobenzene are caused by the chemically inert molecular structure of chlorobenzene.^20^ Pt and Pd nanoparticles are considered to have better sensitization activity; however, Pd is more economical than Pt. Pd nanocatalysts have been used in the hydrodechlorination and oxidation of chlorobenzene and chlorinated VOCs.^21–30^ The catalytic properties of Pd particles combined with ZnO would improve the sensitivity and sensing selectivity toward chlorobenzene; however, the aggregation and growth of Pd particles need to be controlled. The dispersion and stability of Pd loaded on or doped in ZnO are important for improved gas-sensing performance.^31–40^ Because of the aforementioned challenges, literature on the simple and effective fabrication of chlorobenzene-selective Pd@ZnO sensors is scarce.

In the present study, a surface ion exchange method was implemented to decorate porous ZnO nanoplates with Pd nanoparticles. Size-controlled Pd nanoparticles were well dispersed onto ZnO nanoplates, avoiding the surfactants. The samples displayed enhanced response, required a low operating temperature, and exhibited excellent sensing selectivity to chlorobenzene. The explanation for improved sensing performance to chlorobenzene was established after a systematic study.

## Experimental

### Reagents and materials

Analytical-grade zinc acetate dihydrate (Zn(CH_3_COO)_2_·2H_2_O) (≥99.0%), urea (CO(NH_2_)_2_) (≥99.0%), palladium chloride (PdCl_2_) (≥99.99%) and ethanol (≥99.7%) were procured from Sinopharm Chemical Reagent (Shanghai) Co. Ltd. and used without any further treatment and purification. Deionized-water (18.25 MΩ cm) and hydrogen (by hydrogen generator) were self-prepared in laboratory.

### Synthetic methods of porous Pd@ZnO nanoplates

For synthesis of ZnO, 15 mL Zn(CH_3_COO)_2_ solution (0.2 mol L^−1^) was mixed with 15 mL urea solution (0.4 mol L^−1^). The resulting solution was then ultrasonicated for 10 min before being transferred to a Teflon-lined stainless-steel autoclave of 50 mL capacity. Thereafter, it was heated in an oven at 120 °C for 2 h. The sample was then cooled naturally, followed by centrifugation and ethanol/deionized-water washing to collect the precipitate. The sample was dried in air overnight at 60 °C. The as-prepared precursors (hydrozincite [Zn_5_(CO_3_)_2_(OH)_6_]) were annealed at 400 °C for 2 h to create porous ZnO nanoplates.

The impregnation method was used to synthesize Pd^2+^@ZnO (Pd^2+^-adsorbed ZnO) nanocomposites with different Pd^2+^ concentration. ZnO (0.5 g, 6.14 mmol) was suspended in four conical flasks containing 2.72, 8.17, 13.62, and 27.23 mL PdCl_2_ solution (4 g L^−1^) (corresponding molar ratios were 1%, 3%, 5%, and 10%, respectively). The slurries were diluted to 50 mL with water, and the mixtures were illuminated for 12 h under magnetic stirring. Subsequently, the products were obtained by centrifugal separation and labeled as 1% Pd^2+^@ZnO, 3% Pd^2+^@ZnO, 5% Pd^2+^@ZnO, and 10% Pd^2+^@ZnO, respectively.

ZnO decorated with Pd (Pd@ZnO) was synthesized *via* the H_2_ reduction method. The 1% Pd^2+^@ZnO, 3% Pd^2+^@ZnO, 5% Pd^2+^@ZnO, and 10% Pd^2+^@ZnO samples were placed in porcelain boats in a tube furnace, and the samples were treated under H_2_ atmosphere for 2 h at 160 °C. The samples were labeled as 1% Pd@ZnO, 3% Pd@ZnO, 5% Pd@ZnO, and 10% Pd@ZnO, respectively.

### Characterization techniques

Transmission electron microscopy (TEM, HT-7700, Japan) and high-resolution transmission electron microscopy (HRTEM, JEM-2100, Japan) were used for the morphological and structural analysis of the samples. The elemental composition was studied by energy dispersive spectroscopy (EDS) (X-ray microanalyzer built in JEM-2100 microscope). Powder diffraction studies were carried out *via* X-ray diffractometer (XRD) (*λ* = 0.15418 Å, Cu-K_α_ radiation, Bruker D8) for analysis of the crystallinity and phase of the samples. X-ray photoelectron spectroscopy (XPS) (X-ray excitation source, Al-K_α_ (1486.6 eV), Thermo ESCALAB 250, USA) was used to analyze the surface chemical composition and oxidation state. A static system (HW-30A, Hanwei Electronics, China) connected with a computer was used for gas-sensing studies at 30% relative humidity. The BET nitrogen adsorption–desorption graph was recorded using Micromeritics ASAP2020 HD88.

### Gas-sensing measurements

The samples (Pd@ZnO and pure ZnO) were made in paste form using ethanol before being coated onto cylindrical alumina tubes installed with Ni–Cr heating wire. Then, 0.6 mL ethanol was added into 0.05 g Pd@ZnO powder to get a paste in mortar. Subsequently, it was uniformly brushed onto the alumina tubes. The elements were naturally dried and soldered into the gas-sensing component followed by placing the Ni–Cr heating wire, which was used to supply the operating temperature. The sample printed alumina tubes were then aged at 460 °C for 144 h. A microsyringe was used to inject the desired concentration of target gases into the testing chamber. The sensor response value (*R*_s_) is defined as *R*_a_/*R*_g_, where *R*_a_ represents the resistance in air, and *R*_g_ represents the resistance under the target gas.

## Results and discussion

### Structure and morphology

**Fig. 1:**
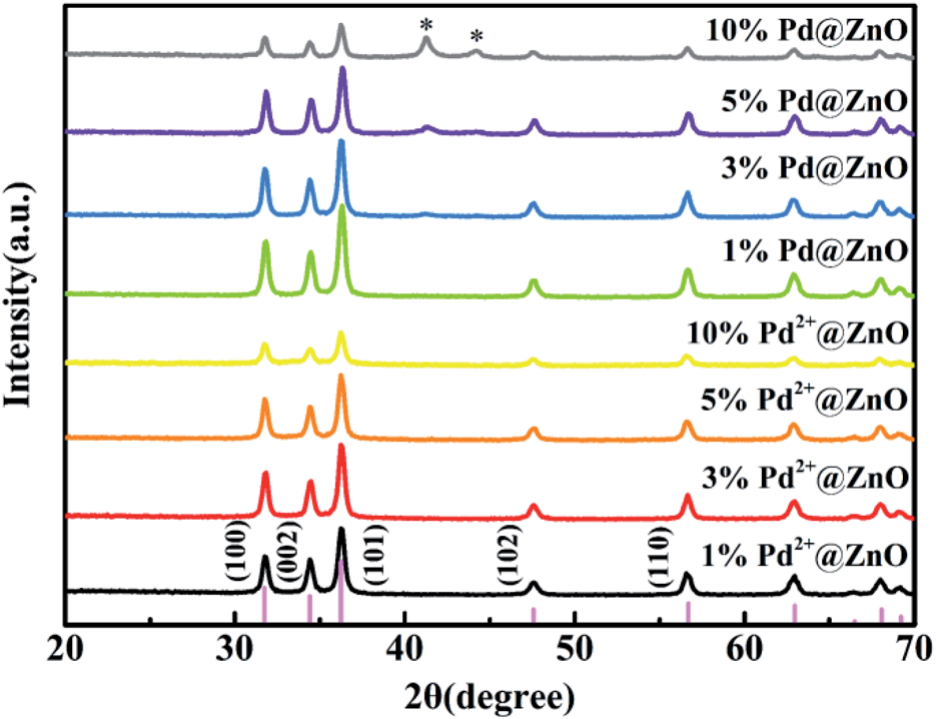
XRD patterns of the synthesized materials: 1% Pd^2+^@ZnO, 3% Pd^2+^@ZnO, 5% Pd^2+^@ZnO, 10% Pd^2+^@ZnO, 1% Pd@ZnO, 3% Pd@ZnO, 5% Pd@ZnO, and 10% Pd@ZnO. The pink bars on the bottom axis stand for the ZnO standard card (JCPDS 36-1451). The asterisks indicate the Pd diffraction pattern peaks.

The XRD patterns (Fig. 1) show that all peaks for 1%, 3%, 5%, and 10% Pd^2+^@ZnO nanoplates match with those of wurtzite ZnO phase, indexed to the card no. 36-1451 of Joint Committee for Powder Diffraction Standards (JCPDS). The crystalline structure of wurtzite ZnO remained intact, as indicated by patterns of Pd^2+^@ZnO and Pd@ZnO materials. This shows that the adsorption of Pd^2+^ and the formation of Pd particles by reduction process did not influence the crystalline structure of ZnO. Before H_2_ reduction, the absence of additional peaks indicates that Pd crystals did not form on the pure ZnO surface. After H_2_ reduction, the XRD patterns of Pd@ZnO display new peaks corresponding to the Pd (111) and (200) lattice planes (JCPDS, no. 46-1043). The intensities of these peaks increased with increasing Pd content, indicating improved crystalline Pd nanoparticles.

**Fig. 2:**
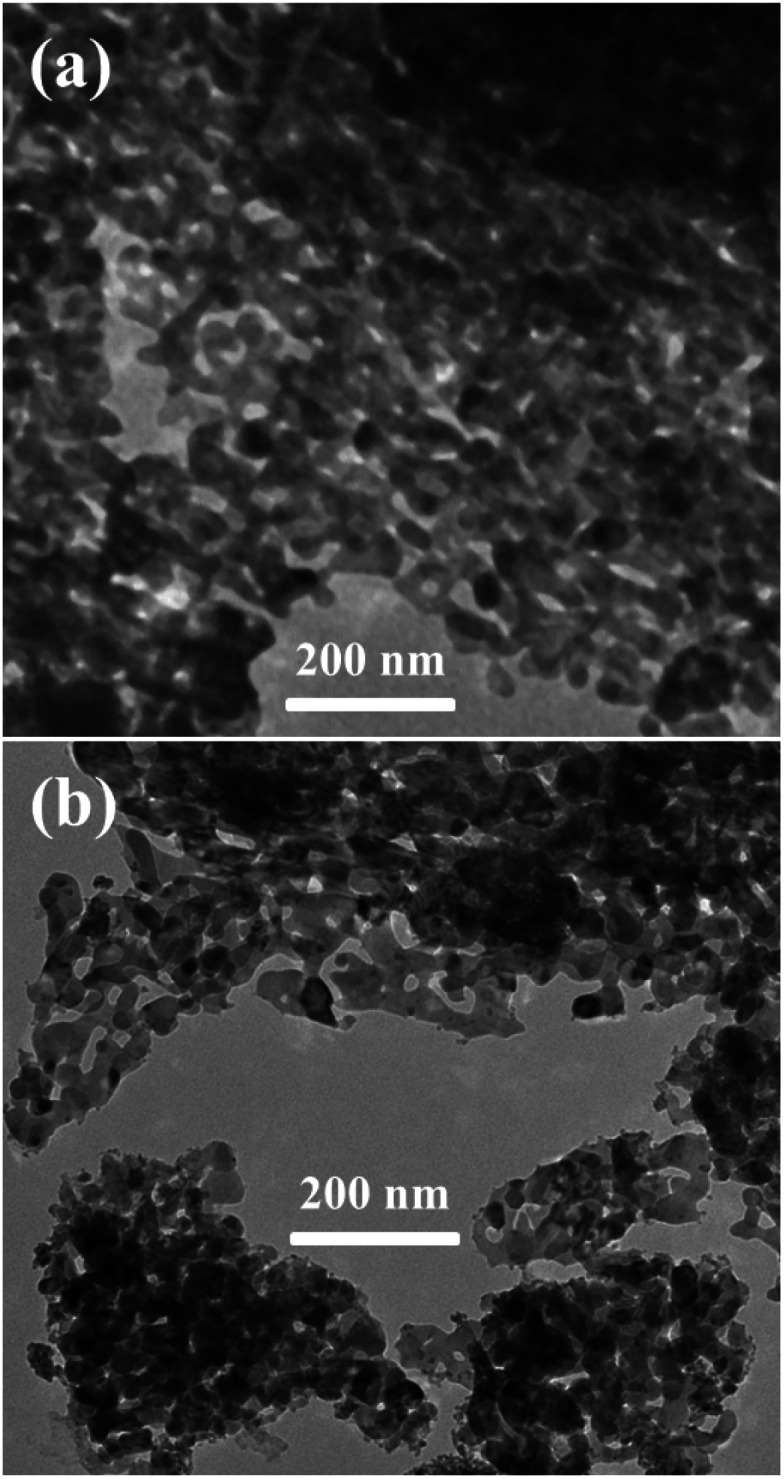
(a and b) TEM images of the pure porous ZnO and 5% Pd@ZnO nanoplates, respectively.

Fig. 2(a) and S1(a)† show TEM images of the porous structure of pure ZnO nanoplates. Lattice fringe spacing is approximated to 0.28 nm, in accordance with the (100) crystal plane of wurtzite ZnO, as revealed by the HRTEM image of porous ZnO nanoplates (Fig. S1(b)†). According to literature,^10,14,39,41^ when the (100) facet is greatly exposed in porous and single crystalline ZnO nanosheets, it displays superior gas-sensing performance. The inclusion of Pd^2+^ did not modify the structure of ZnO in the Pd^2+^@ZnO nanocomposites as there was no additional crystal formation (Fig. S1(c)–(f)†). This characterization is in agreement with the XRD results. The amounts of elemental Pd in 1% and 3% Pd^2+^@ZnO products are less than 0.5%, but in 5% and 10% Pd^2+^@ZnO products are 0.9 ± 0.1% and 1.0 ± 0.1%, as analyzed by EDS spectra (Fig. S1(g)–(j)†). It is very likely that the addition of Pd^2+^ has a limit and approaches adsorption saturation on the ZnO surface.

**Fig. 3:**
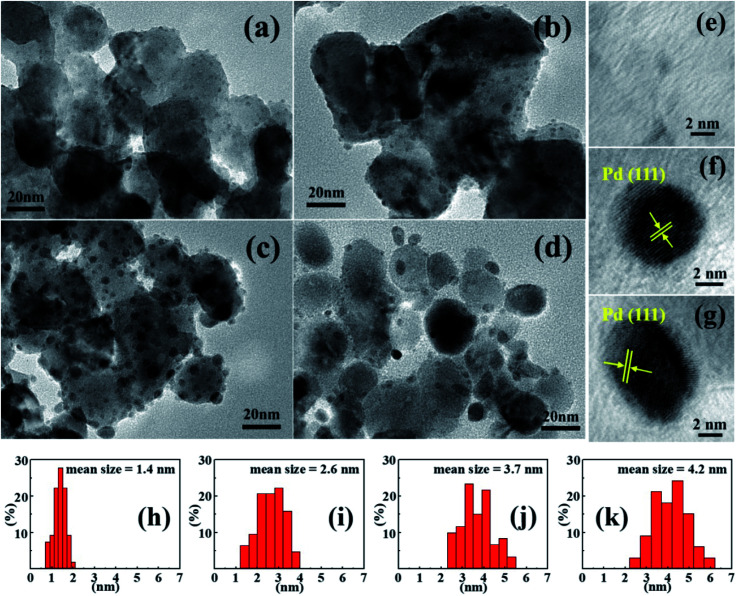
(a–d) TEM images of 1% Pd@ZnO, 3% Pd@ZnO, 5% Pd@ZnO, and 10% Pd@ZnO, respectively. (e–g) HRTEM images of Pd particles in the 1% Pd@ZnO, 5% Pd@ZnO, and 10% Pd@ZnO, respectively. (h–k) Pd particle size distribution of 1% Pd@ZnO, 3% Pd@ZnO, 5% Pd@ZnO, and 10% Pd@ZnO, respectively.

As shown in Fig. 2(b), small Pd particles appear to be well dispersed on porous ZnO nanoplates after H_2_ reduction. HRTEM images of the irregular edge domains of porous nanostructures are shown in Fig. 3(a)–(d). With increasing amount of PdCl_2_, the size of Pd particles is observed to increase. When the ZnO surface adsorbs more Pd^2+^, more Pd nucleating sites form in micro-areas, which is more conducive to the growth of Pd particles under the same conditions.^42^ The fringe patterns obtained from HRTEM images (Fig. 3(e)–(g)) show a separation of 0.22 nm in agreement with the (111) lattice spacings of cubic Pd. Through statistical measurement, the calculated average sizes are about 1.4, 2.6, 3.7, and 4.2 nm for Pd particles of 1%, 3%, 5%, and 10% Pd@ZnO products, respectively, as shown in Fig. 3(h)–(k). These results show that the Pd nanoparticles are size-controlled and well-dispersed on porous ZnO nanoplates.

In surface defects and zinc vacancies, the Zn^*n*+^ cations (ionic radius 0.74–0.88 Å) are not in tetrahedral coordination and possess an ionic radius similar to Pd^2+^ cations (ionic radius 0.86 Å).^43^ Pd^2+^ cations can replace exposed Zn^*n*+^ cations and also be stably present in the oxygen coordination environment. The surface ion exchange in the impregnation procedure promotes the uniform and stable adhesion of Pd^2+^ cations in the material surface.^16^ The H_2_ reduction and calcination generate the crystallization and growth of Pd. Pd nanoparticles can be stably attached onto ZnO by this simple preparation method, effectively avoiding particle aggregation and growth.

### Surface species and valence state

**Fig. 4:**
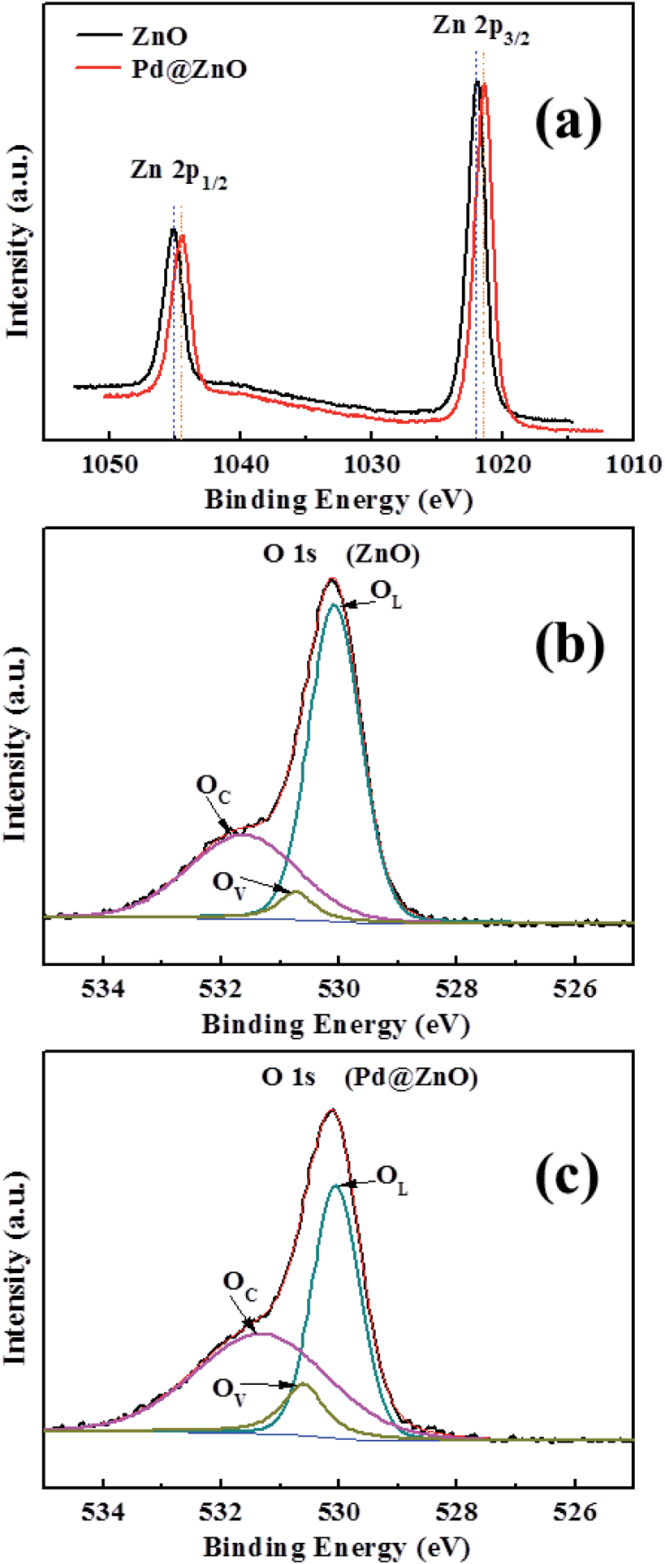
(a) Zn 2p XPS spectrum plots of 5% Pd@ZnO and pure porous ZnO nanoplates; (b and c) O 1s XPS spectrum plots of 5% Pd@ZnO and pure porous ZnO nanoplates, respectively.

Elemental composition and valence state analysis were conducted using XPS (Fig. 4 and S2†), with binding energy value *ε* ≤ ±0.01 eV. Fig. 4(a) shows that the Zn 2p spectrum plot of pure ZnO has binding energies at 1045.12 and 1021.91 eV, corresponding to Zn 2p_1/2_ and Zn 2p_3/2_, respectively, which also tallies with other reported results.^44,45^ Nanocomposites of 5% Pd@ZnO showed negative shifts of binding energies of 1044.49 and 1021.43 eV. These are attributed to the formation of Pd–Zn intermetallic compounds, which are formed on the Pd@ZnO material surface after the surface ion exchange and reduction treatment. A high temperature treatment over 300 °C promotes the development of PdZn alloys.^46–49^ However, the H_2_ reduction in the proposed method did not generate well-crystallized PdZn alloys, resulting in the formation of a small amount of intermetallic compounds on the surface of Pd@ZnO nanomaterials, resulting in the lack of PdZn characterization in the XRD analysis. The Pd 3d spectra of 5% Pd^2+^@ZnO and 5% Pd@ZnO were identified using XPS (Fig. S2†). The peaks near 342.36 and 336.75 eV are assigned to Pd^2+^ species with a slight positive shift to the literature, which confirms the entrance of Pd^2+^ cations into the surface lattice by ion exchange.^21,37^ After calcination with H_2_ reduction, the binding energies of Pd^0^ species were approximately 340.56 and 335.28 eV. A positive shift of approximately 0.27 eV appeared in binding energies of both Pd 3d_3/2_ and 3d_5/2_, compared with 340.29 and 335.01 eV of pure Pd, suggesting that interactional Pd with Zn increased the Pd 3d binding energy as a consequence of charge transfer and rehybridization.^47,50^

Fig. 4(b) and (c) shows the O 1s pure porous ZnO spectra and 5% Pd@ZnO nanoplates, respectively. The O 1s XPS peak can be divided into profiles of three main species through Gaussian fitting: surface lattice oxygen of ZnO (O_L_), surface oxygen vacancies (O_V_), and dissociated or chemisorbed oxygen species on the surface (O_C_).^37,41^ The binding energies of these oxygen species are given in Table 1. Negative shifts are observed in the O_L_, O_V_, and O_C_ peaks of Pd@ZnO samples relative to those of pure ZnO. The result indicates that all the surface oxygen species will acquire the transferred electrons, which make their reduction easier.^16^ Further, the relative percentages of the O_V_ and O_C_ species in Pd@ZnO samples are significantly increased, suggesting a higher capability to absorb active oxygen species compared to pure ZnO. These results can be used to understand the influence of Pd modification on gas-sensing performance of porous ZnO nanoplates.

**Table 1:** XPS results of O 1s spectra of pure ZnO nanoplates and 5% Pd@ZnO by Gaussian fitting^a^

Samples	O_L_	O_V_	O_C_
**ZnO nanoplates**			
Binding energy (eV)	530.08	530.75	531.64
*ε* ≤ ±0.01 eV			
Relative percentage (%)	61.1	5.1	33.8

**Pd@ZnO nanoplates**			
Binding energy (eV)	530.05	530.61	531.31
*ε* ≤ ±0.01 eV			
Relative percentage (%)	42.6	10.4	47.0

a O_L_ represents surface lattice oxygen of ZnO; O_V_ represents surface oxygen vacancies; O_C_ represents dissociated or chemisorbed oxygen species on the surface.

### Gas-sensing performance

**Fig. 5:**
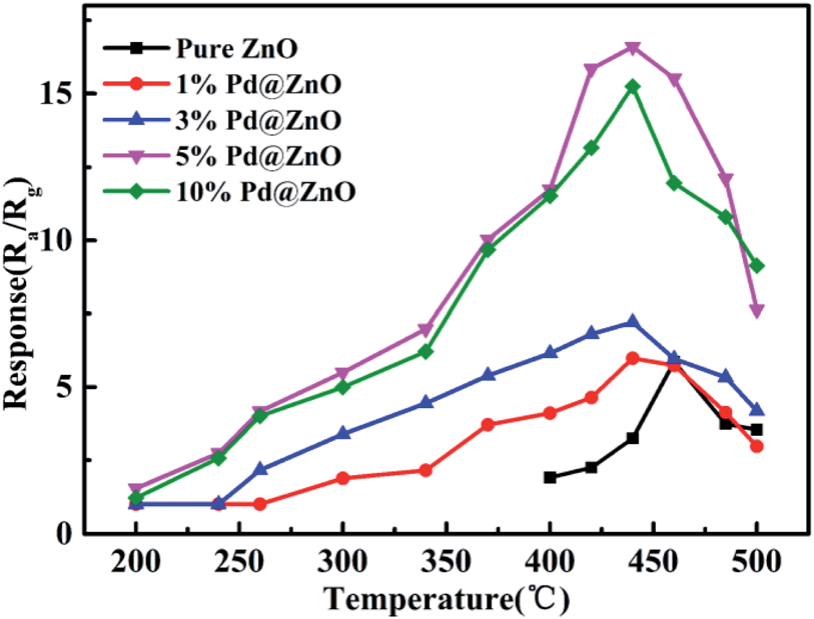
Response plots of the obtained sensors: 1% Pd@ZnO, 3% Pd@ZnO, 5% Pd@ZnO, 10% Pd@ZnO, and pure ZnO nanoplates toward 100 ppm chlorobenzene at different operating temperatures.

The response of as-prepared sensors to chlorobenzene at various operating temperatures was investigated. The results of 1% Pd@ZnO, 3% Pd@ZnO, 5% Pd@ZnO, 10% Pd@ZnO, and pure ZnO sensors are shown in Fig. 5. The range of operating temperature was from 200–500 °C, and the target gas concentration was 100 ppm. At 200 °C, the ZnO sensor displays no response, and all the four Pd@ZnO sensors exhibit weak responses to chlorobenzene. All the four Pd@ZnO sensors display increasing responses with ascending operating temperature up to 440 °C, and then it decreases. However, the highest response of pure ZnO nanoplates to chlorobenzene appears at 460 °C. Comparing with pure ZnO sensor, all of the Pd@ZnO sensors display lower optimum temperature at 440 °C and have better responses than that of pure ZnO sensor before 440 °C, indicating that the decoration of Pd enhances the sensitivity to chlorobenzene. It is worth noting that the initial response of 5% Pd@ZnO sensor appears obviously at 240 °C, moreover, the response value of 5% Pd@ZnO sensor at 300 °C nearly equals the maximum response value of pure ZnO sensor at 460 °C; therefore, the Pd modification has significantly reduced the operating temperature of the gas-sensing test. This means that energy consumption can be effectively reduced in practical applications. However, to facilitate the study of subsequent experiments, all further tests were performed at the optimal temperature of 440 °C, at which 5% Pd@ZnO sensor was shown to exhibit better performance with increasing Pd content, even exceeding that of 10% Pd@ZnO sensor, which may be due to the more appropriate particle size (approximately 3–4 nm) with higher sensitization activity. Hence, 5% Pd@ZnO sensor was considered as the object of study with optimal performance and was used in following studies.

**Fig. 6:**
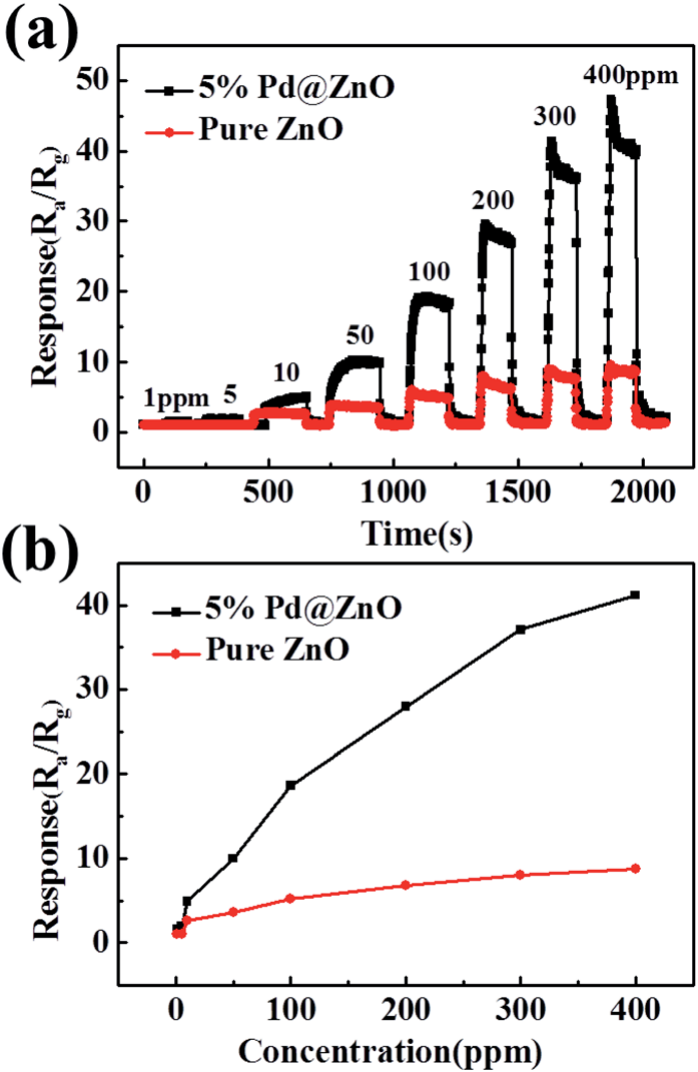
(a) Typical dynamic response plots of 5% Pd@ZnO and pure ZnO sensors toward chlorobenzene with increasing concentration at 440 °C; (b) the dependent responses of 5% Pd@ZnO and pure ZnO sensors on chlorobenzene concentration at 440 °C.

Fig. 6(a) shows typical dynamic response plots of 5% Pd@ZnO and pure ZnO sensors at 440 °C toward chlorobenzene. The plots display a stepped growth with increasing chlorobenzene concentration. Fig. 6(b) displays the responses of 5% Pd@ZnO sensor to chlorobenzene at a good detectable concentration range from 1 to 400 ppm at 440 °C. The responses of the Pd@ZnO sensor are higher and increase faster than those of the pure ZnO sensor, indicating that the 400 ppm concentration of the target gas did not approach adsorption saturation. The sensor response displays a non-linear concentration dependence, which can be attributed to the kinetics of gas molecules in terms of the adsorption, oxidation, and desorption processes on the surface of the semiconductor sensor. The concentration and chemical structure of the gas species influence the kinetics.^51,52^

After the gas is introduced into the chamber, the time consumed to attain 90% of the stabilized response value is defined as the response time. After the gas is withdrawn from the chamber, the time required for a 90% reduction from the original response value is referred to as the recovery time.^16^ Taking the cyclic curve at 100 ppm for example (Fig. S3†), the response and recovery times of 5% Pd@ZnO sensor to chlorobenzene were 19 and 7 s, respectively, which are longer than those of pure ZnO sensor (5 and 4 s, respectively). The detection speed of the proposed sensor is acceptable for practical applications.

**Fig. 7:**
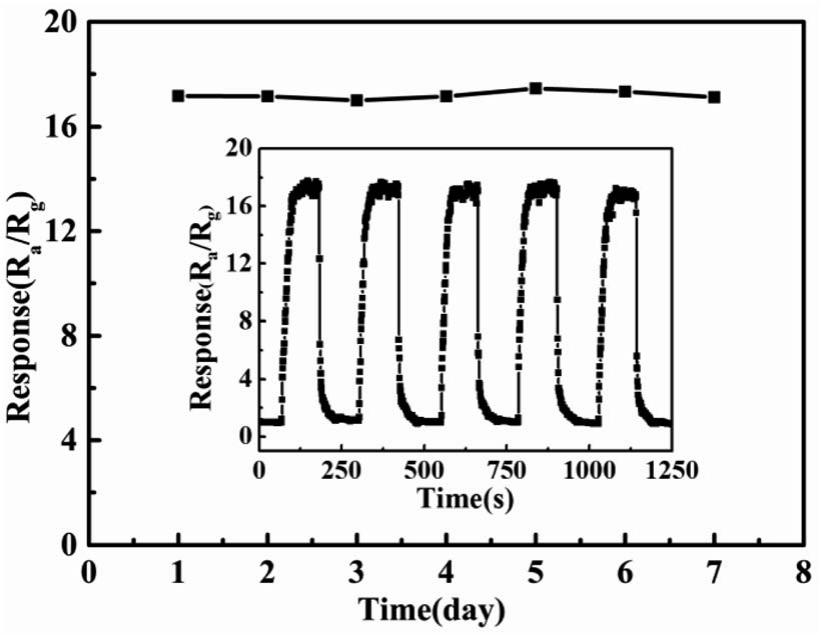
Stability study for 5% Pd@ZnO sensor towards 100 ppm chlorobenzene at 440 °C during a week (the inset exhibits the repeatability under the same conditions).

Fig. 7 shows the responsiveness of 5% Pd@ZnO sensor to chlorobenzene at 440 °C during one week, revealing good stability. The inset figure shows the reproducibility under the same conditions, which demonstrates that the sensor retains its original response value in repeated experiments. Good stability and reproducibility are beneficial to practical applications. Furthermore, the relative humidity (RH) effect on 5% Pd@ZnO sensor response was also probed. Fig. S4† shows that the sensor performance is influenced by different relative humidity. The responses to chlorobenzene ascend with increasing RH value, up to the maximum at the RH value of 50%, and then they descend. The fluctuation of the RH effect on the sensor response results from the competitive adsorption between H_2_O and target gas molecules on the sensor surface.^53^ When the RH value exceeds 50%, H_2_O molecules adversely affects the chlorobenzene adsorption.

**Fig. 8:**
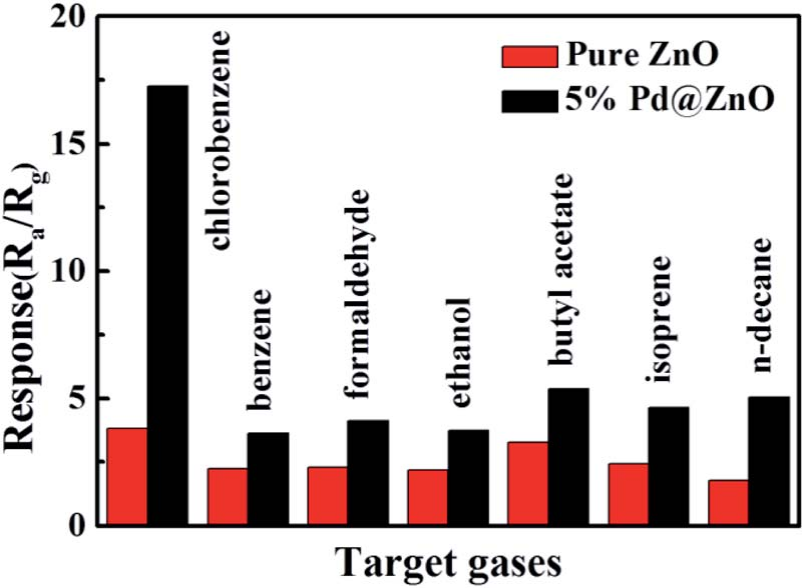
Response histogram of 5% Pd@ZnO (black) and pure ZnO (red) sensors toward 100 ppm chlorobenzene, benzene, formaldehyde, ethanol, butyl acetate, isoprene, and *n*-decane at 440 °C.

Selectivity is an important factor in the applicability of the metal oxide gas sensor, which can avoid a mistaken alarm and offer extensive usage. VOCs generally contain seven groups: halogenated hydrocarbons, aromatic hydrocarbons, aldehydes, oxy hydrocarbons, esters, terpenes, and aliphatic hydrocarbons.^16,53^ To explore the selectivity of pure ZnO and 5% Pd@ZnO sensors, six other typical VOCs gases (benzene, formaldehyde, ethanol, butyl acetate, isoprene, and *n*-decane) were also tested as a contrast in Fig. 8, which together with chlorobenzene represent seven groups of VOCs gases. All the analytic gases were of 100 ppm concentration and detected at 440 °C. It is evident that the responses of pure ZnO sensor to all the target gases are lower than 5, along with poor selectivity. Nevertheless, 5% Pd@ZnO sensor shows a higher sensitivity to all the target gases than those of pure ZnO sensor, in particular, the highest response value to chlorobenzene (17.4 ± 0.4) is nearly 4.5 times that of pure ZnO sensor (3.8 ± 0.1). The response to chlorobenzene is markedly higher than those to other target gases, implying that 5% Pd@ZnO sensor is a practical candidate for selectively detecting chlorobenzene gas. In addition, more target gases have been tested, for instance, the response values are 3.5 ± 0.1 (Pd@ZnO sensor)/3.0 ± 0.1 (ZnO sensor) to methanol and 4.0 ± 0.1 (Pd@ZnO sensor)/2.8 ± 0.1 (ZnO sensor) to hexaldehyde, *etc.*, which also show enhanced sensitivity and chlorobenzene-selectivity of 5% Pd@ZnO sensor. This gas-sensing selectivity may result from the unique catalytic activity of Pd. Pd nanocatalysts have been used for the efficient oxidation of chlorobenzene and chlorinated organics.^28–30,54^ Pd^0^ can absorb O_2_ to form active [Pd^2+^O^2−^] species, which oxidize the absorbed chlorobenzene, and the Pd^2+^ is simultaneously reduced to Pd^0^.^54^ The catalytic selectivity results because the rise in temperature favors the oxidation of chlorobenzene rather than its chlorination, and the oxidation rate of polychlorobenzenes is faster than their formation rate over 400 °C.^28–30^ Moreover, 1,2-dichlorobenzene and 1,3-dichlorobenzene were also tested as target gases under the same conditions (Fig. S5†). Compared with pure ZnO sensor, 5% Pd@ZnO sensor possesses clearly enhanced gas-sensing responses to various types of chlorobenzene compounds.

### Gas-sensing mechanism

**Fig. 9:**
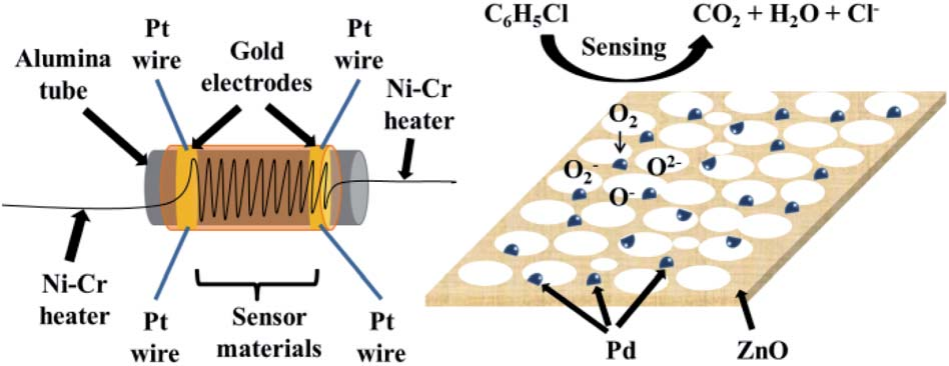
Schematic illustration of the gas sensor structure and the highly dispersed size-controlled Pd nanoparticles on ZnO nanostructures for gas-sensing applications.

A schematic illustration of the obtained gas sensor structure, composition of the materials used in the gas sensor, and general mechanism is shown in Fig. 9. A more detailed mechanism of the special gas-sensing performance needs further clarification. The specific surface areas of porous ZnO and Pd@ZnO nanoplates can influence the gas-sensing performance,^16,53^ which is calculated from the nitrogen adsorption–desorption isotherm data *via* the Brunauer–Emmett–Teller (BET) method (Fig. S6†). The surface area of 5% Pd@ZnO nanoplates (12.94 ± 0.01 m^2^ g^−1^) was lower than that of pure ZnO nanoplates (23.28 ± 0.01 m^2^ g^−1^), and the average pore size of 5% Pd@ZnO nanoplates (approximately 75 nm) was larger than that of pure ZnO nanoplates (approximately 35 nm). The changes indicate that the deposition of Pd nanoparticles by H_2_ reduction at high temperature caused certain destruction of the porous lamella surface structure. Hence, the improved gas-sensing properties of Pd@ZnO are attributed to Pd decoration.

The adsorption–oxidation-desorption process governs the gas-sensing mechanism and depends on the surface depletion layer to some extent. The pure and modified ZnO gas sensors have free electrons on the surface, which are trapped by the oxygen molecules in air to generate oxygen species (O^2−^, O^−^, and O_2_^−^). This leads to the formation of the electron depletion layer.^16,17,37^ When the gases for study are inserted into the test chamber, the sensor surface adsorbs gas molecules, which are oxidized by the oxygen species (O^2−^, O^−^, and O_2_^−^).

Compared to pure ZnO sensor, the enhanced sensing properties of Pd-modified sensors resulted from the combination of chemical sensitization mechanism and electronic sensitization mechanism. The chemical sensitization aroused by interfacial atom transport and the electronic sensitization originating from interfacial electronic redistribution resulted in two routes for the sensing reaction.^17,37,40^ For Pd-modified ZnO, the oxygen molecules were reduced preferentially by Pd nanoparticles to form oxygen anions in air, which subsequently overflowed to the ZnO lattice surface. When the gases were adsorbed onto the surface of Pd nanoparticles, gas molecules were activated by Pd and subsequently migrated to react with oxygen species on the ZnO surface, thus increasing the surface conductivity (known as chemical sensitization).^37,40^ In addition, the direct reaction between target gases and the surface oxygen species caused increased surface conductivity (known as electronic sensitization), due to the electron depletion layers, forming a continuous band resulting from Pd heterogeneous hybrids.^17,37^ The two sensitization effects of Pd contributed to the sensing of Pd@ZnO materials toward chlorobenzene.

More importantly, through the analysis of the O 1s XPS peak, more active oxygen species are created in virtue of the small-sized Pd particles. In addition, the activated and ZnO-supported Pd catalysts are powerful for the catalytic oxidation of chlorinated aromatics. The high temperature over 400 °C makes the oxidation rates of polychlorinated by-products faster than the chlorination rates of chlorinated aromatics,^28–30^ altering the intrinsic interaction between Pd and Cl, which avoids causing polychlorinated by-products. The nature of ZnO support also plays a key role in the orientation of the sensing oxidation reaction *via* the synergetic effect between Pd and the support.^29,30^

Table S1† shows the chlorobenzene-sensing comparation among results obtained in this work and reported in the literature. Combined with the above factors, Pd-modified porous ZnO nanoplate sensor has exhibited excellent sensing performance from the higher responses, good selectivity, lower operating temperatures, good stability, and reproducibility.

## Conclusions

Well-dispersed and size-controlled Pd nanoparticles were modified on porous ZnO support *via* surface ion exchange and subsequent H_2_ reduction procedures for selectively sensing chlorobenzene. Approximately 3–4 nm sized and highly active Pd particles were scattered and stabilized on ZnO support. A small amount of Pd–Zn intermetallic compounds were also generated on the Pd@ZnO nanocomposite surface. Pd@ZnO sensors displayed superior sensitivity compared to pure ZnO sensor in the gas-sensing tests. The XPS characterization confirmed more catalytic sites and oxygen species generated by Pd@ZnO hybrids toward the adsorbed chlorobenzene molecules. The operating temperature could be decreased from 460 °C of ZnO sensor to 300 °C of 5% Pd@ZnO sensor, with almost the same sensitivity. The optimal 5% Pd@ZnO sensor showed maximum response, which was about 4.5 times that of pure ZnO sensor to chlorobenzene at the optimum operating temperature of 440 °C. Although chlorobenzene owns chemically inert molecular structure, good gas-sensing selectivity was also attained. It is ascribed that the supported Pd catalysts are efficient for the catalytic oxidation of chlorinated aromatics, as well as the synergetic effect between Pd and the support in the gas-sensing reaction. In addition, fine gas-sensing stability and repeatability were attained. This work provides a new strategy for controlled heterogeneous Pd@ZnO nanocomposites and the further development of high-efficiency gas sensors toward chlorobenzene compounds.

## Conflicts of interest

There are no conflicts to declare.

## Supplementary Material

RA-009-C9RA09705H-s001
