# Supplementary material for: Well-dispersed Pd nanoparticles on porous ZnO nanoplates via surface ion exchange for chlorobenzene-selective sensor
Source: RSC Adv. 2019 Dec 20;9(72):42351–9. doi: 10.1039/c9ra09705h (PMC9076696; doi:10.1039/c9ra09705h)
Supplement: RA-009-C9RA09705H-s001 [file RA-009-C9RA09705H-s001.pdf]

Electronic Supplementary Material (ESI) for RSC Advances.
This journal is © The Royal Society of Chemistry 2019
Please do not adjust margins

ARTICLE

Received 00th January 20xx,
Accepted 00th January 20xx

DOI: 10.1039/x0xx00000x

Well-dispersed Pd nanoparticles on porous ZnO nanoplates via surface ion exchange for chlorobenzene-selective sensor

Zhenyu Feng,*[a] Cuiling Gao,[b] Xicheng Ma[a] and Jinhua Zhan[a]

Figure legends

Fig. S1. TEM images and EDS spectra of 1% Pd^{2+}@ZnO, 3% Pd^{2+}@ZnO, 5% Pd^{2+}@ZnO, and 10% Pd^{2+}@ZnO, and HRTEM image of porous ZnO nanoplate

Fig. S2. Pd 3d XPS spectra of 5% Pd^{2+}@ZnO and 5% Pd@ZnO

Fig. S3. Response/recovery curves of 5% Pd@ZnO and pure ZnO sensors towards chlorobenzene

Fig. S4. Responses of 5% Pd@ZnO sensor toward chlorobenzene in different relative humidity

Fig. S5. Responses of 5% Pd@ZnO and pure ZnO sensors toward chlorobenzene, 1,2-dichlorobenzene, and 1,3-dichlorobenzene

Fig. S6. Nitrogen adsorption-desorption isotherms of 5% Pd@ZnO and pure porous ZnO nanoplates with BJH pore size distribution insets

Table S1. Chlorobenzene-sensing comparation among results obtained in this work and reported in the literature

[a.] *Key Laboratory for Colloid and Interface Chemistry of Education Ministry, School of Chemistry and Chemical Engineering, Shandong University, Jinan, Shandong, 250100, P. R. China.*
[b.] *Shandong Institute for Product Quality Inspection, Jinan, Shandong, 250102, P. R. China.*
* Corresponding author. E-mail: fengzhenyu@sdu.edu.cn; Tel: +86 53188363768.

Please do not adjust margins

Fig. S1

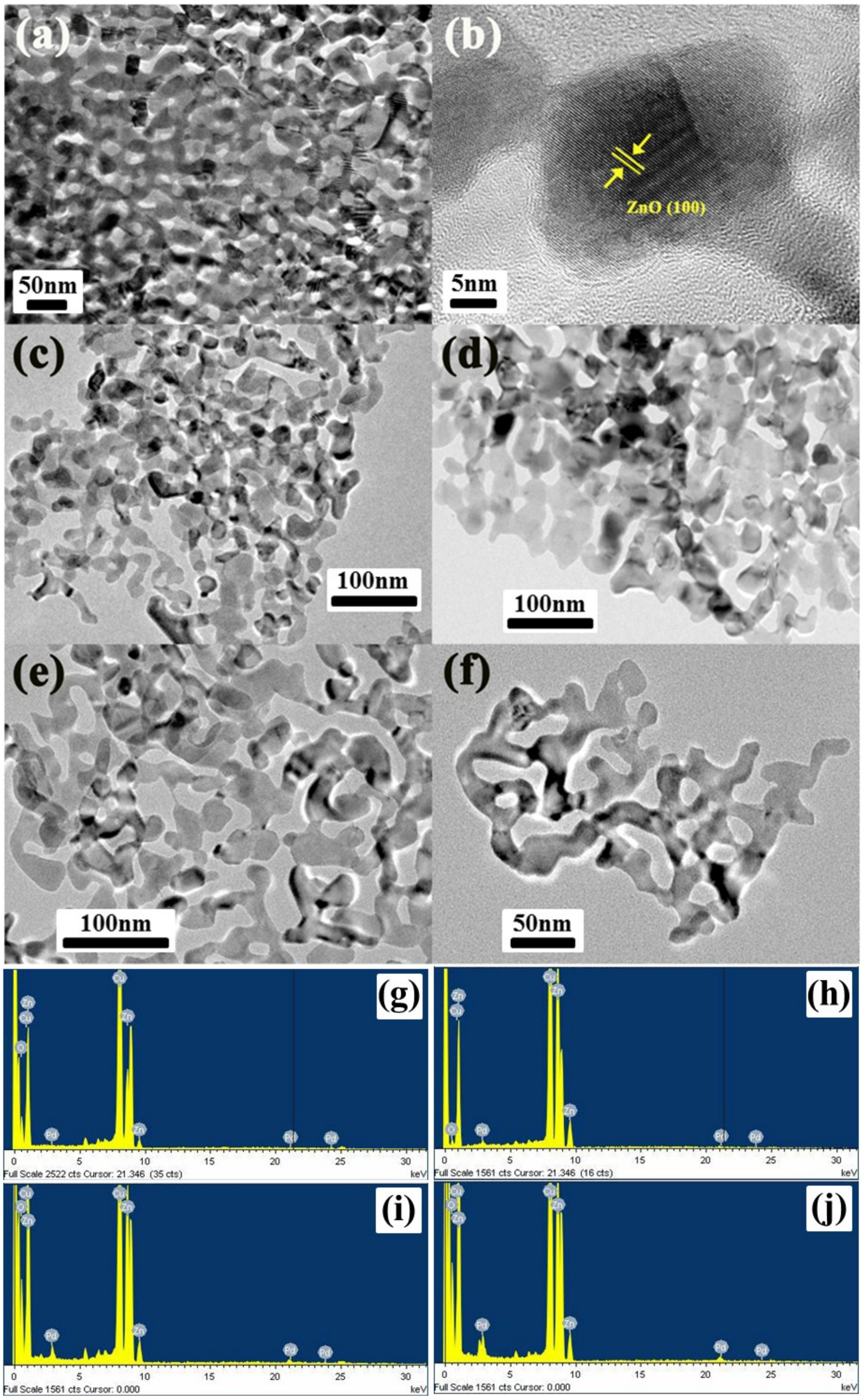

Fig. S1. (a), (c), (d), (e), and (f) represent TEM images of the pure ZnO, 1% Pd^{2+}@ZnO, 3% Pd^{2+}@ZnO, 5% Pd^{2+}@ZnO, and 10% Pd^{2+}@ZnO nanoplates, respectively. (b) shows HRTEM image of the pure ZnO nanoplate. (g), (h), (i), and (j) represent EDS spectra of the 1% Pd^{2+}@ZnO, 3% Pd^{2+}@ZnO, 5% Pd^{2+}@ZnO, and 10% Pd^{2+}@ZnO, respectively.

Please do not adjust margins

Fig. S2

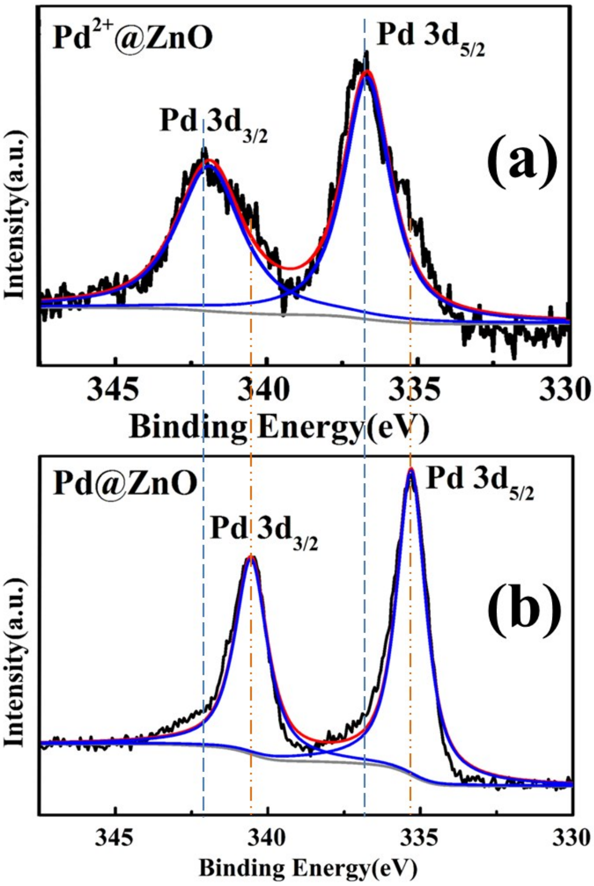

Fig. S2. Pd 3d XPS spectra of the obtained materials: (a) 5% Pd^{2+}@ZnO and (b) 5% Pd@ZnO.

Please do not adjust margins

Fig. S3

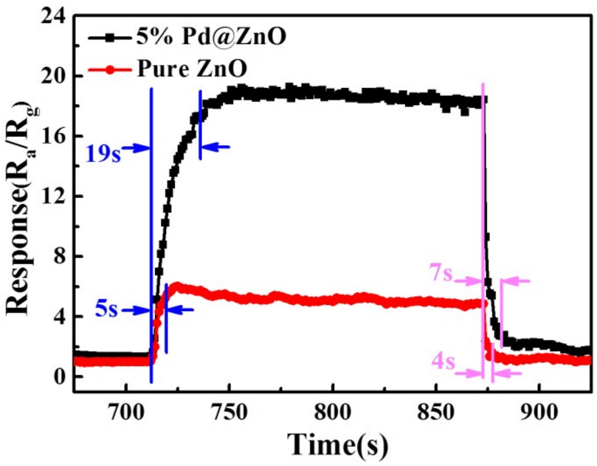

Fig. S3. Response/recovery curves of 5% Pd@ZnO and pure ZnO sensors towards chlorobenzene with 100 ppm at 440 °C.

Please do not adjust margins

Fig. S4

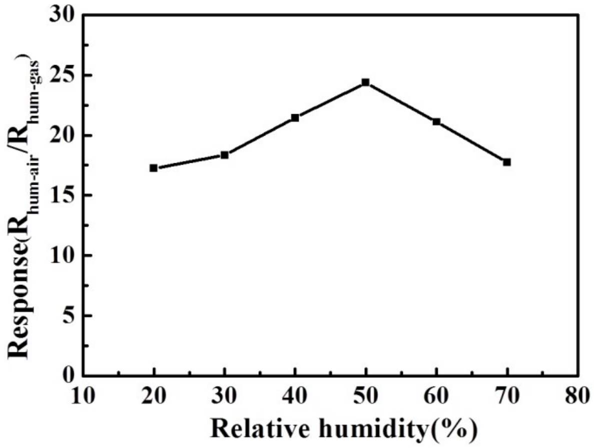

Fig. S4. Responses of 5% Pd@ZnO sensor toward 100 ppm chlorobenzene in different relative humidity at 440 °C. The response is defined as the ratio of sensor resistance ($R_{hum-air}$) in humid air to that ($R_{hum-gas}$) in humid air with 100 ppm chlorobenzene.

Please do not adjust margins

Fig. S5

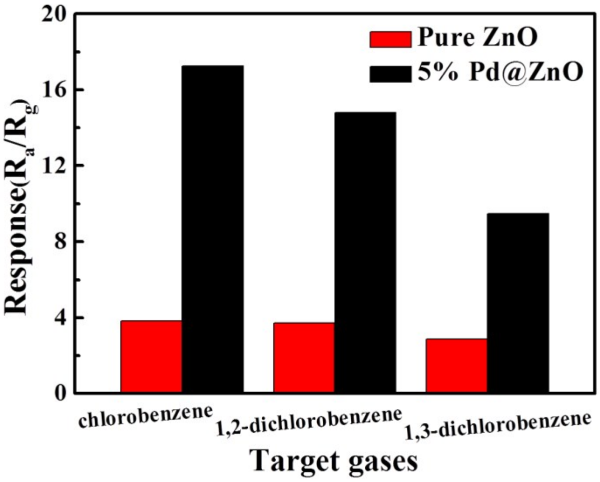

Fig. S5. Responses of 5% Pd@ZnO (black) and pure ZnO (red) sensors towards 100 ppm chlorobenzene compounds (gases) including chlorobenzene, 1,2-dichlorobenzene and 1,3-dichlorobenzene at 440 °C.

Please do not adjust margins

Fig. S6

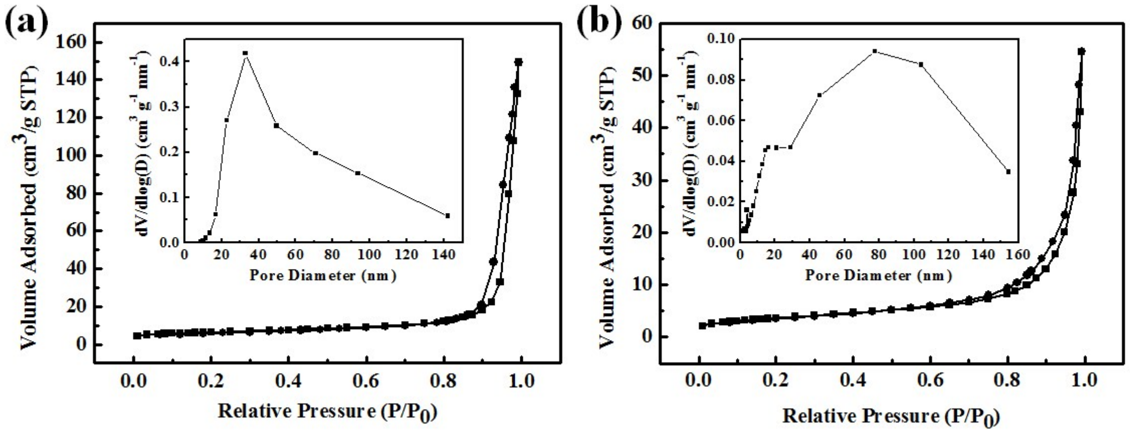

Fig. S6. Nitrogen adsorption-desorption isotherms with BJH pore size distribution insets of (a) pure porous ZnO nanoplates, (b) 5% Pd@ZnO. According to the IUPAC classification, the hysteresis loops corresponding to H3 type reveal the feature of mesoporous material. The specific surface areas of (a) and (b) are 23.28±0.01 and 12.94±0.01 m^2/g, respectively.

Please do not adjust margins

Table S1. Chlorobenzene-sensing comparation among results obtained in this work and reported in the literature.

Sensor materials	Initial response temperature (°C)	Detection limit	Selectivity study	Response/recovery time (s)	Ref.
Pd@ZnO	240	1 ppm	Yes	19/7	This work
Prussian blue film	25	24 ppm	Yes	18/12	[1]
Pt-decorated porous single-crystalline ZnO nanosheets	200	30 ppb	Yes	20/10	[2]
In-doped coral-like SnO_2 nanostructures	170	0.2 ppm	-	6.7/25.8	[3]
In_2O_3 nanorods and nanoparticles	270	10 ppm	-	-	[4]
Au-deposited porous single-crystalline ZnO nanoplates	260	2 ppm	-	10/14	[5]
Porous ZnO nanoplates	150	-	-	103/22	[6]

Based on different equipment and testing conditions, various gas-sensing materials show their features in chlorobenzene detection. The sensor materials in this work have enhanced response and selectivity compared to controlled ZnO sample, which also have acceptable chlorobenzene-sensing performance compared to results reported in the literature.

References:

[1] T. X. Fu, *Analytical and Bioanalytical Chemistry*, 2011, **401**, 1167.

[2] C. P. Gu, H. H. Huang, J. R. Huang, Z. Jin, H. X. Zheng, N. Liu, M. Q. Li, J. H. Liu and F. L. Meng, *Sensors and Actuators A*, 2016, **252**, 96.

[3] Y. T. Wan, J. Y. Liu, W. Li, F. L. Meng, Z. Jin, X. Y. Yu, X. J. Huang and J. H. Liu, *Nanotechnology*, 2011, **22**, 315501.

[4] Y. J. Tang and J. M. Ma, *RSC Advances*, 2014, **4**, 25692.

[5] X. Han, Y. Sun, Z. Y. Feng, G. C. Zhang, Z. C. Chen and J. H. Zhan, *RSC Advances*, 2016, **6**, 37750.

[6] Z. H. Jing and J. H. Zhan, *Advanced Materials*, 2008, **20**, 4547.

Please do not adjust margins

Please do not adjust margins
